# Vegetable and Animal Food

**Published:** 1846-06

**Authors:** 


*Vegetable and Animal Food.*—We learn from a late number of the Gazette Médicale, that a very economical application has been made of the recent discoveries in organic chemistry, by the substitution of a vegetable for an animal diet in hospitals. Some enterprising French chemist has lately announced that beans and peas contain more gelatin, fibrin and fat (!) and in general a greater amount of nutriment, than beef, veal, and mutton! The authorities of the hospital at Avignon, acting upon this view, and with the most anxious solicitude for the well-being of the patients, have cut them off from the debilitating diet of mutton and beef; and, upon chemical principles, have ordered them a more stimulating and nutritious kind of food, in the shape of beans and peas! The presence of a large quantity of *fat* in these vegetables has also enabled them to reduce materially the consumption of olive oil!

Strange as it may appear to those acquainted with modern chemical doctrines, and the accuracy of analysis, the patients have most ungratefully and unfeelingly objected to the change. An antileguminous spirit has sprang up among them, and they persist in declining to be fed upon chemical theory. With the plain facts before them, that chemistry has proved that animal and vegetable substances contain identical proteinaceous compounds, they are unreasonable enough to declare that they prefer mutton and beef! How the matter will end it is impossible to say, but we are informed, that in spite of the watchfulness of the hospital attendants, the antileguminous party have actually succeeded in privately smuggling into the institution those provisions which have been denounced as deficient in nutritive matter.—*Ibid.*

